# Formulation and antibacterial properties of lollipops containing of chitosan- zinc oxide nano particles on planktonic and biofilm forms of *Streptococcus mutans* and *Lactobacillus acidophilus*

**DOI:** 10.1186/s12903-023-03604-9

**Published:** 2023-12-01

**Authors:** Hamideh Sadat Mohammadipour, Parastoo Tajzadeh, Mahshid Atashparvar, Samira Yeganehzad, Maryam Erfani, Fatemeh Akbarzadeh, Sima Gholami

**Affiliations:** 1 Restorative and Cosmetic Dentistry, Dental Research Center, School of Dentistry, Mashhad University of Medical Sciences, Mashhad, Iran; 2 Kashmar School of Medical Sciences, Mashhad University of Medical Sciences, Mashhad, Iran; 3 Mashhad University of Medical Sciences, Mashhad, Iran; 4 Department of Food Processing, Research Institute of Food Science and Technology (RIFST), Mashhad, Iran; 5 Radiology Department, Razavi International Hospital, Mashhad, Iran; 6 Department of Chemistry, Faculty of Basic Sciences, Islamic Azad University, Mashhad, Iran; 7 Department of Restorative and Cosmetic Dentistry, School of Dentistry, Mashhad University of Medical Sciences, Mashhad, Iran

**Keywords:** *Streptococcus mutans*, *Lactobacillus acidophilus*, Anti-caries lollipop, Dental caries, Chlorhexidine, Nano chitosan, Zinc oxide

## Abstract

This study aimed to formulate and characterize the experimental lollipops containing chitosan- zinc oxide nanoparticles (CH-ZnO NPs) and investigate their antimicrobial effects against some cariogenic bacteria. The CH-ZnO NPs were synthesized and characterized by X-ray diffraction (XRD), Fourier Transform Infrared Spectroscopy (FTIR) analysis, and Transmission electron microscope (TEM). Then, four groups were made, including lollipops coated with 2 and 4 ml of CH-ZnO NPs, 0.7 ml CH-ZnO NPs incorporated lollipops, and those with no CH-ZnO NPs. Their antibacterial effectiveness against *Streptococcus mutans* and *Lactobacillus acidophilus* was evaluated by direct contact test and tissue culture plate method in planktonic and biofilm phases, respectively. Chlorhexidine mouthrinse (CHX) was used as a positive control group. In the planktonic phase, the antibacterial properties of both groups coated with CH-ZnO NPs were comparable and significantly higher than incorporated ones. There was no significant difference between CHX and the lollipops coated with 4 ml of NPs against *S. mutans* and CHX and two coated groups against *L. acidophilus*. None of the experimental lollipops in the biofilm phase could reduce both bacteria counts. The experimental lollipops coated with 2 and 4 ml of CH-ZnO NPs could reveal favorable antimicrobial properties against two cariogenic bacteria in the planktonic phase.

## Introduction

Despite significant advancements in the field of dentistry and the widespread availability of basic preventive treatments in many regions across the globe, dental caries remains a prevalent chronic disease, particularly among children [1]. While dental caries is a complex, multifactorial condition, there is a well-established direct link between fermentable carbohydrates and their development [2]. *Streptococcus mutans* and *Lactobacillus acidophilus* are recognized as the primary culprits in the initiation and progression of dental caries, as they metabolize simple carbohydrates like sucrose, leading to the production of organic acids, most notably lactic acid [3–5]. These acids accumulate within the biofilm’s fluid phase, resulting in a drop in pH levels, which, in turn, leads to demineralization and superficial enamel erosion [3].

To combat dental caries, various preventive approaches have been explored, encompassing the use of fluoride or calcium-phosphate-containing products, antimicrobial agents like chlorhexidine mouthwash (CHX), and probiotics, all of which have proven effective in reducing caries development or progression [6–10]. Adding antimicrobial agents into edible products, such as candies, lollipops, and gums, which may prove more appealing to patients, especially children, and encourage greater consumption, thereby harnessing the benefits of antimicrobial ingredients [10]. Notably, the efficacy of sugar-free lollipops containing licorice extract in reducing salivary *S. mutans* count has shown promise as an effective measure, particularly when consumed twice daily, offering significant reductions over a three-month period, especially in high-caries-risk children [11–14].

Chitosan (CH), a biomaterial derived from the deacetylation of chitin, mainly sourced from marine shell waste, has garnered attention in dentistry due to its myriad properties, including indigestibility, biocompatibility, biodegradability, non-toxicity, muco-adhesion, and a broad spectrum of antimicrobial and antifungal activity [15–18]. CH, either alone or in combination with other agents in the form of mouthwash, chewing gum, or toothpaste, has proven effective in reducing gingival inflammation, plaque accumulation, adhesion, and salivary *S. mutans* counts, while also mitigating demineralization and promoting remineralization [19–24].

Recent research has highlighted that chitosan nanoparticles outperform chitosan particles in terms of muco-adhesion, permeability, antimicrobial properties, and anti-tumor effects [24–26]. Specific functional groups within the CH structure enable bonding with various elements, such as metals or metal oxides [27]. Zinc oxide nanoparticles (ZnO NPs) have emerged as promising candidates in the biomedical field, owing to their biocompatibility, low toxicity for humans, and cost-effectiveness due to their increased specific surface area and enhanced particle surface activity [28, 29]. Combining CH with ZnO nanoparticles yields a synergistic blend with robust biomedical and antibacterial properties, increasing CH’s solubility in the process [27, 30]. In the dental arena, this composite has demonstrated potent antibacterial effects against both gram-positive and gram-negative bacteria, including *S. mutans* and *L. acidophilus* [31].

Given the preference of patients, especially those at high risk of dental caries, for edible products, the incorporation of antibacterial agents into sugar-free candies and lollipops presents a practical means of reducing bacterial accumulation and adhesion, ultimately mitigating caries. This approach may be particularly beneficial in low and middle-income countries with high rates of childhood caries, often attributable to elevated sugar consumption [13, 32, 33]. Thus, our in vitro study is designed to formulate and characterize experimental lollipops containing CH-ZnO nanoparticles (CH-ZnO NPs) and investigate their antimicrobial effects on both planktonic and biofilm forms of *S. mutans* and *L. acidophilus*. We have set forth the null hypothesis that these experimental lollipops do not exhibit any antimicrobial effects on S. mutans and L. acidophilus in both planktonic and biofilm phases.

## Materials and methods

This in vitro study was conducted at the Department of Cosmetic and Restorative Dentistry (Mashhad University of Medical Sciences, Mashhad, Iran). The study protocol was approved by the local ethics committee of Mashhad University of Medical Sciences, Iran (IR.MUMS.DENTISTRY.REC.1399.119).

The research was carried out in three main phases. At first, CH-ZnO nanocomposite were prepared and the characterization of it was confirmed by several tests. The experimental lollipops containing 0.7 ml of CH-ZnO nanocomposite or coated with 2 and 4 ml of CH-ZnO nanocomposite were made. After that, the antimicrobial properties of experimental lollipops in two planktonic and biofilm phases were investigated. The complete description of these phases is presented as follows.

The materials employed in the present study and their respective manufacturer are presented in Table 1.

**Table 1:** Materials were used in this study and the manufacturers’ information

	Materials	Manufacturer
Materials for making experimental nano composite	ZnO	Merke, Damstadt, Germany
	Chitosan	Mohandesin Moshaver Bartar, Mashhad, Iran
	Citric acid	Merke, Damstadt, Germany
	Acetic acid	Merke, Damstadt, Germany
	NaOH	Merke, Damstadt, Germany
Materials for making experimental lollipops	Isomalt	BENEO, Mannheim, Germany
	Xanthan	Persian Gum Tam, Mashhad, Iran
Materials for antibacterial assessment	MRS Agar culture medium	Ibresco life sciences, Italy
	MRS Broth culture medium	Ibresco life sciences, Italy
	BHI-Agar culture medium	Ibresco life sciences, Italy
	BHI-Broth culture medium	Ibresco life sciences, Italy
	Gas pack	Soroush, Tehran, Iran
	Chlorhexidine mouthrinse	Shahredaru, Tehran, Iran
	*Streptococcus mutans* (IBRC10682)	Iranian Biological Resource Center, Tehran, Iran
	*Lactobacillus acidophilus* (IBRC10815)	Iranian Biological Resource Center, Tehran, Iran

### Preparation of CH-ZnO nanocomposite

To produce the CH-ZnO nanocomposite, the simultaneous sedimentation method was used in this study. To prepare CH-ZnO composite, the CH polymer chains should be expanded to absorb the ZnO particles. Thus 0.5 mM ZnO (Merke) was dissolved in 100 ml of 1% acetic acid solution at 55 ° C. Then 1 g of CH was added to the mixture and was stirred (Universal-AlfaD500, Iran) until a homogenous and transparent mixture was obtained.

The polymeric form was reproduced by the gradual addition of 0.1 M of NaOH (Merk). The obtained white-colored sediment was stirred at laboratory temperature, and the pH was set at 10. When CH and ZnO are dissolved in dilute acetic acid (Merk), Zn ^2+^ is formed by regulating the solution’s acidity. At pH = 10 the unstable compound, Zn(OH)^2^, is formed. The OH- and NH^2^ in CH can bond with the metal. Therefore, a stable complex of CH-ZnO NPs was formed as pH reaches ten by adding the NaOH.

Finally, to neutralize the alkaline pH from the addition of NaOH, the product was thoroughly rinsed with distilled water and placed in an ultrasonic bath (Digital ultrasonic cleaner, CD,4820, China) for 30 min to homogenize ions dispersion. The final solution was mixed in a water bath with a high-speed mixer in a constant round at 65 ° C for four hours. The film was then removed, dried at 50 C for 24 h, and crushed to form a homogenous powder.

### Characterization of NPs

#### FTIR spectroscopy

Fourier transform-Infrared spectroscopy (FTIR) (AVATAR 370, Thermo nicolet Corp., Waltham, Massachusetts, United States) was used to identify the structure and chemical bonds, functional groups, and possible interactions between the components. The chemical bonds created between the 400–4000 cm^− 1^ wavelength range were evaluated. The material being mixed with a KBr pellet. In a typical experiment 2–3 mg of the compound was mixed with the KBr and ground to a powder. The KBr pellets were obtained using hydraulic pressure of 7–10 psi. The discs were scanned in the 400– 4000 cm^− 1^ range to obtain FTIR spectra.

#### X-ray diffraction pattern (XRD) test

XRD at an ambient condition was used to evaluate the structure of the samples. The crystal structure of the samples was evaluated by X-ray diffraction (EXL2, Philips, Amsterdam, Netherland) with copper (Cu-Kα1 line, λ = 1.54056 Cm^− 1^) at a diffraction angle (2θ) range between 5˚ and 80˚. Powder sample was compressed between two smooth glass films and the XRD analysis was in dispersion 2 angles of 5°– 80° at a step size of 0.02° with 2 s/step as scanning rate using 5 a voltage 45 kV, a Ni-filtered Cu K radiation (= 1.5406 A)° and a filament current of 40 mA.of.

#### Transmission electron microscopy (TEM evaluation)

The nanoparticles’ morphology, shape and size was investigated by Transmission electron microscope (TEM) (CEM 902 A, Zeiss, Jena, Germany).

### The lollipops preparation

The experimental lollipops were incorporated and coated with CH-ZnO nanocomposite and mainly based on isomalt material. Since 1 gr CH and 0.05 gr ZnO were dissolved in 120 ml liquid containing acetic acid and NaOH, each ml of the final solution contained 8.75 mgr of CH-ZnO nanocomposite. However, due to several washing and high temperature, the exact concentration of CH-ZnO NPs in the final solution was lower than this amount. The CH-ZnO nanocomposite were added to the lollipops in two approaches:

1. The coated lollipops (Groups 1 and 2): In this approach, at first, 0.3 gr xanthan powder (Persian Gum Tam Company) was dissolved in 30 ml sterile distilled water to prepare a solution containing 1% wt of xanthan solution. This solution was mixed for 2 h and was then stored in the refrigerator for 24 h. Afterward, 0.5 ml and 2 ml of Xanathan solution were mixed with 2 ml and 4 ml CH-ZnO-containing solution, respectively, and put in an ultrasonic device (Ultrasonic CD-4820, Shenzhen Codyson Electrical Ltd., Guangdong, China) for favorable particle dispersion. Isomalt powder was melted and delivered to a glass slab to create granules with a maximum diameter of 0.5 cm. After cooling, the prepared granules were immersed in the solutions containing xanthan and CH-ZnO nanocomposite.
2. The CH-ZnO NPs incorporated lollipops (Group 3): In this approach, the isomalt powder (BENEO) was melted and poured into silicone molds. Then, 0.7 ml of CH-ZnO nanocomposite solution was added to each mold and mixed until the homogeneous suspension mixture was obtained. For the primary antimicrobial analysis, the flavoring agents did not add to the mixture. Molds were cooled in a refrigerator for 24 h.

Based on earlier studies’ data, the minimum biofilm eradication concentration (MBEC) of CH against *S. mutans* was 5 mg/ml. Thus 0.7 ml of a solution containing CH-ZnO NPs with 6.125 mg of CH-ZnO nanocomposite was added to each lollipop (4–6 gr). This minimum amount was obtained based on several preparations that did not interfere with the lollipop formation process.

Finally, the samples obtained from these two approaches were transferred to the microbiology laboratory to evaluate the antibacterial properties.

### Antibacterial assessment

#### Preparation of culture media

The antibacterial evaluation of experimental lollipops was done in planktonic and biofilm formation phases. At first, the standard solutions and microbial suspensions, as well as culture media, were prepared as follows:

The 0.5 McFarland standard solution: To prepare 0.5 McFarland standard, 0.5 ml of 0.48 M barium chloride was mixed with 99.5 ml of 0.36 N sulfuric acids. This medium contained 1.5 × 10^8^ CFU/mL bacteria and can be restored for 6 months in a dark environment. If any signs of sediment were seen, the medium was not usable.

The 0.5 McFarland bacterial suspension: To make bacterial suspension, 3 to 4 colonies from the upper cultured colonies were separated and suspended in a physiological saline tube to obtain turbidity equal to the McFarland tube. After isolating the bacteria, some bacterial colonies were removed by inoculating loops and dissolved in sterile saline to create 0.5 McFarland microbial suspension. Because in the antibiogram test, the amount of turbidity is significant, so the correct number of bacteria should be selected, and more or less than half of McFarland should not be removed. If the turbidity is less than half a McFarlane, dissolve some other sample in sterile physiological saline, or if the opacity is more than half McFarland, in this case, some sterile physiological serum should be added to reach a suitable turbidity equal to half McFarland.

Culture media preparation: For harvesting *S. mutans* bacteria, the Brain Heart Infusion (Ibresco life sciences) in two forms of solid (BHI-A) and liquid (BHI-B) were used. To obtain one liter of solid and liquid medium, 52.8 and 37 g/L of distilled water were dissolved, and heated to a boil using Bunsen burner and completely dissolve the medium, respectively. Then the pH of the solution was set, and the solutions were sterilized.

*L. acidophilus* was cultured in MRS medium in both solid (MRS-A) and liquid (MRS-B) types. To prepare one liter of the solid medium, 68.2 g/L of distilled water and 52.2 g/L of distilled water for the liquid medium was dissolved and heated. After complete dissolution and pH adjustment, culture media were sterilized by an autoclave.

The solid culture media (Ibresco life sciences) were poured into plates, and the liquid media (Ibresco life sciences) were poured into tubes and stored in the refrigerator after cooling.

#### Planktonic stage

The direct Contact Test (DCT) was used to evaluate the antibacterial properties in the planktonic phase. The bacteria used for assessment of antibacterial activity, including *S. mutans* (IBRC10682) and *L. acidophilus* (IBRC10815), were cultured in a brain-heart infusion (BHI) broth (Ibresco life sciences, Italy) to produce turbidity of 0.5 McFarland (equivalent to 1.5 × 10^8^ colony-forming unit (CFU)/mL).

The prepared incorporated and coated lollipops were placed in sterile laboratory tubes and sterilized. Then 10 µL of microbial suspension was applied on the surface of each sample and remained for 1 h. According to the defined surface and slope, after adding 10 µL of suspension, the same contact was created. All samples were monitored by the researcher every time. After that, 240 µL of BHI culture medium for *S. mutans* and MRS-B for *L. acidophilus* were added to the test tubes. So, 1 min, 10 µL of the resultant mixture was seeded on the solid BHI-A and MRS-A cultures for *S. mutans* and *L. acidophilus*, respectively, and spread over the medium culture through the floating technique using a sterile steak hook.

**Fig. 1:**
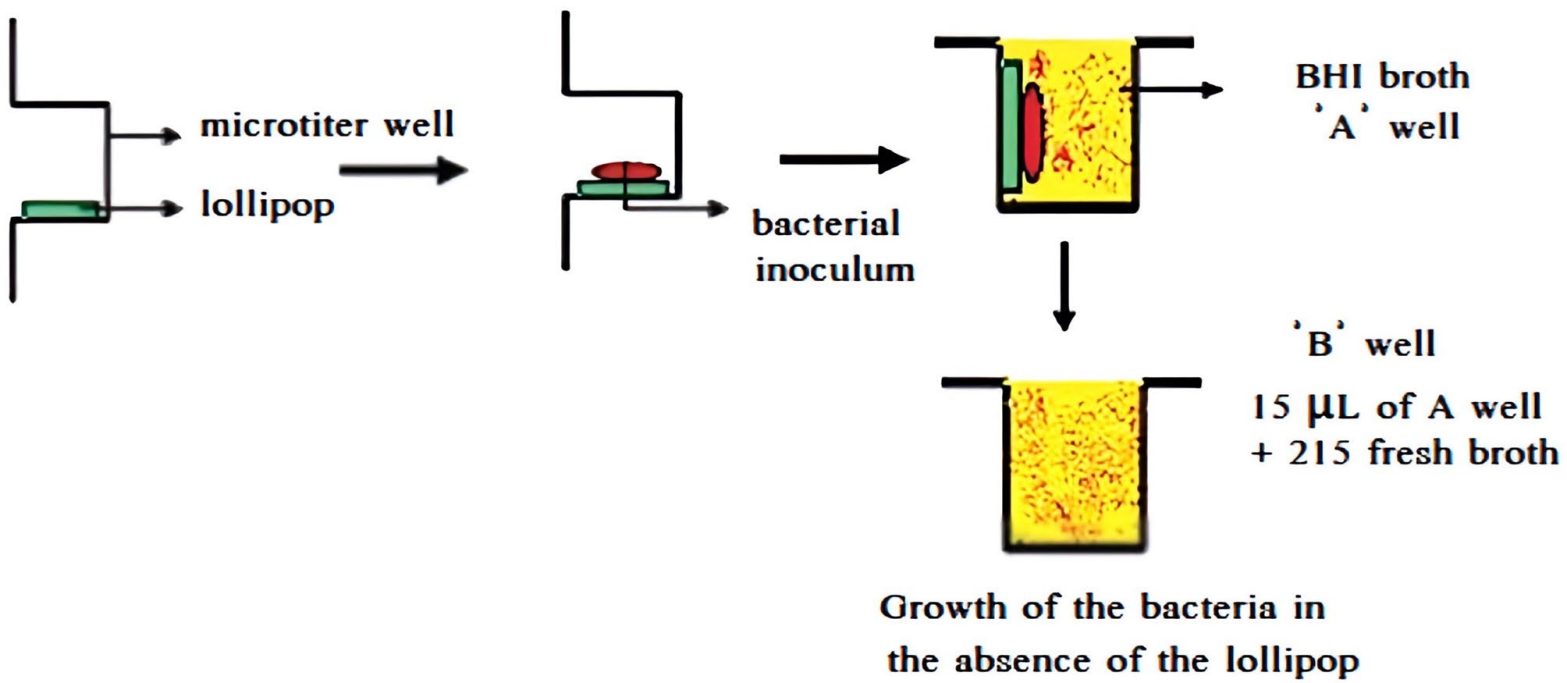
Schematic view of direct contact test for evaluating the lollipops’ antibacterial properties by counting colony-forming units

After incubating the plates (Fine TechSSl- 202) at 37◦C for 24 h (in a 5% CO2, aerobic atmosphere for *S. mutans*), the colonies were counted using a colony counter device (Mey med, South Korea) and reported as colony-forming units (CFUs/ml) (Fig. 1). Each test (plate) was counted 3 to 5 times, and the average was recorded as the final report.

In order to confirm the studied bacteria during the tests, microbial smears were stained using Gram staining. Biochemical and microbiological tests such as catalase, oxidase, and fermentation of mannose and sorbitol sugars were also used.

#### Biofilm stage (tissue culture plate test for bacterial biofilm phase)

To evaluate the antibacterial activity in the biofilm phase, 190 µl of each medium containing 0.5 McFarland turbidity was poured into a 96-well plate. Ten µl of new bacterial suspension with a concentration of 0.5 McFarland was added to wells one to four. In the fifth well, sterile distilled water was poured instead of bacterial suspension and incubated at 37 ° C for 24 h. Then, the inserted contents were removed from each well under a sterile hood and washed three times with distilled water to remove media, suspension, and free-floating cells. At this stage, the bacteria could form a biofilm on the plate wall, except in the fifth well, where no bacterial suspension was used.

The solid lollipop was crushed and placed at the bottom of the tube. Then the wells were filled as follows:

**Well 1 (positive control)**

The culture medium and CHX (Shahredaru, Tehran, Iran).

**Well 2**

The culture medium and coated lollipop granules with 2 ml of CH-ZnO NPs solution.

**Well 3**

The culture medium and coated lollipop granules with 4 ml of CH-ZnO NPs solution.

**Well 4**

The culture medium containing 0.7 ml of CH-ZnO NPs solution.

**Well 5 (negative control)**

The culture medium with no microbial suspension and lollipops.

After this step, 200 µL of 0/025% Safranin solution was added to each well for two min to color the formed biofilms. After 2 min, the Safranin was drained and washed with distilled water three times. Then, 200 µL of the ethanol-acetone solution (50% V/V) was added to each well for 15 min. In this step, the Safranin dyes that had entered the biofilm were released, and the color was read at the 492 nm wavelength by an ELISA device (Convergent, EL-Reader 96x). The more biofilm was formed, the more dye was absorbed, and after adding an acetone-alcohol solution, it produced more dye, and finally, more adsorption was recorded by ELISA. The following formula was used to calculate the potency of CH-ZnO NPs in killing biofilm cells [34]:

$$\text{Reduction Percent =}\left[\frac{\left(c-b)-(t-b\right)}{\left(c-b\right)}\right] \times 100$$

**Fig. 2:**
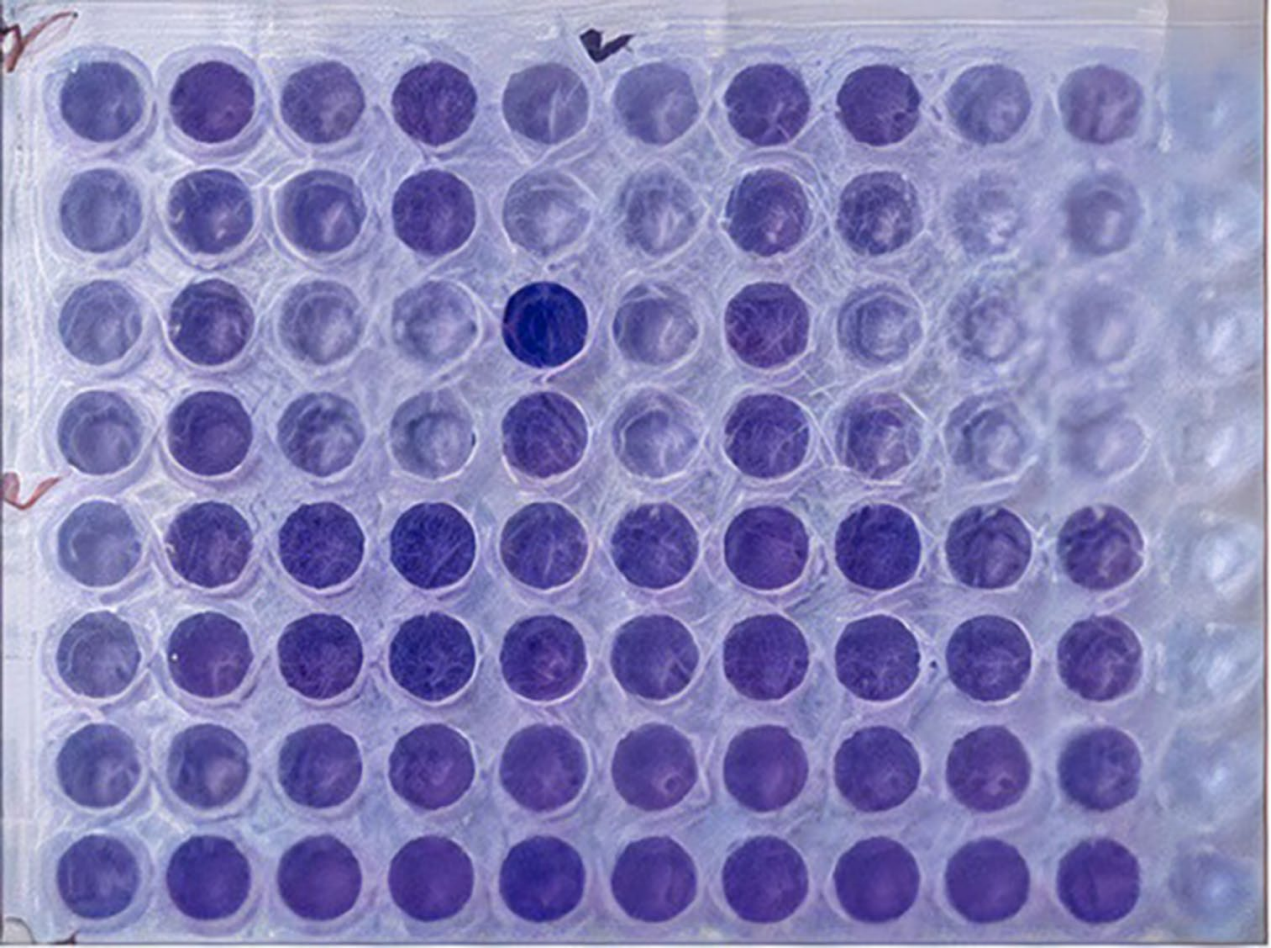
Preparing tissue culture plate for evaluating the biofilm phase

In this formula, b presents optical density (OD) for positive control wells, c is OD for negative control wells, and t is the OD of the wells exposed to CH-ZnO NPs. These steps were performed for both *S. mutans* and *L. acidophilus*. The tissue culture plate was prepared for antibacterial evaluation in the biofilm phase, as presented in Fig. 2.

### Statistical analysis

Data were analyzed using the SPSS statistical software (version 22.0, IBM). The Kolmogorov-Smirnov test was used to assess the normality of µSBS data distribution. One-way ANOVA was also used for comparing the antimicrobial properties of study groups. When the differences between groups were statistically significant, the One-Sample t-Test was used to show the differences between them. All the statistical analyses were performed with the significance level set at 5%.

## Results

### Preparing CH-ZnO nanocomposite

When CH and ZnO were dissolved in diluted acetic acid, by regulating the solution acidity, Zn^2+^ ions were created, and in pH = 10, the unstable combination Zn (OH)^2^ was created.

Zn^2+^ + 2OH^-^ <=˃ Zn (OH)^2^.

The -NH2 and -OH- functional groups of CH could create a coordinated bond with metal in pH = 10, and a stable complex of CH-ZnO NPs was created.

### CH-ZnO nanocomposite characterization

#### FTIR spectroscopy

**Fig. 3:**
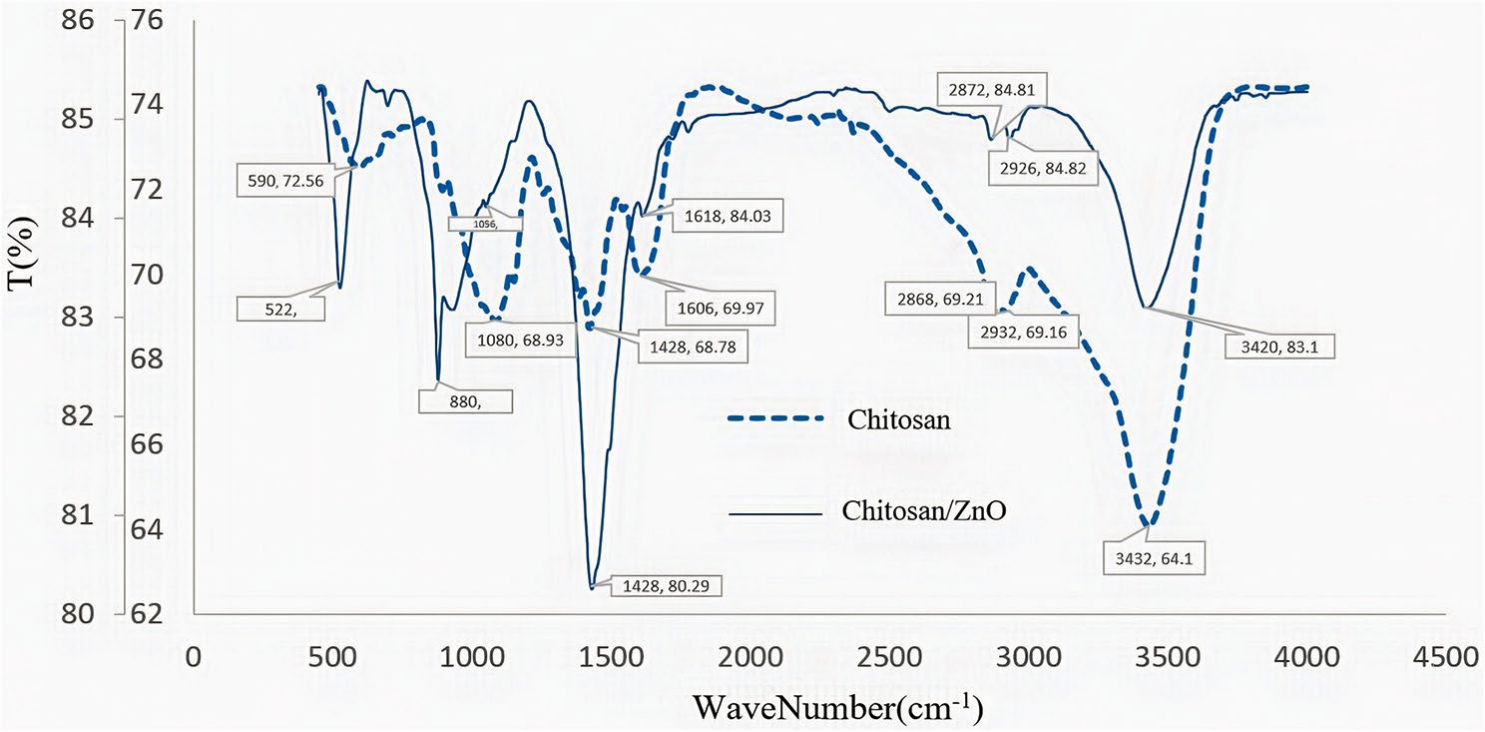
FTIR spectrum of the CH-ZnO NPs and CH

The results from FTIR spectroscopy regarding the functional groups and chemical bonds in molecules in the presence and absence of ZnO NPs are presented in Table 2; Fig. 3.

**Table 2:** The Results of FTIR test

Assignment	Wave Number (cm^− 1^)	
	Matrix	Matrix + ZnO NPs
C-Cl stretching vibration	590.72	522.38
C-O stretching	1080.68	1056.84
-C-H bending	1428.68	1428.80
Carboxyl group	1606.69	1618.84
-CH2 stretching	2868.69	2872.84
C-H stretching	2932.69	2926.84
OH-/N-H stretching vibration	2432.64	3420.83

As shown in Fig. 3, the sharp absorption peaks were observed at 3423 cm-1, 2932 cm-1, 2868 cm-1, and 1080 cm-1 wavelengths for (N-H) -(O-H), C-H, CH2, and C-O bonds, respectively. The sharp peak at 1618 cm-1 wavelength, which is the characteristic peak of the amide group, indicated the hydrogen-tensile bond between C = O and COO groups. Compared to CH, the CH-ZnO curve had sharper peaks at lower wavelengths, indicating a strong interaction between these groups and ZnO. In the complex curve, a new absorption peak was observed at 880 cm-1, which may be related to the flexural adsorption of the N-H bond.

**Fig. 4:**
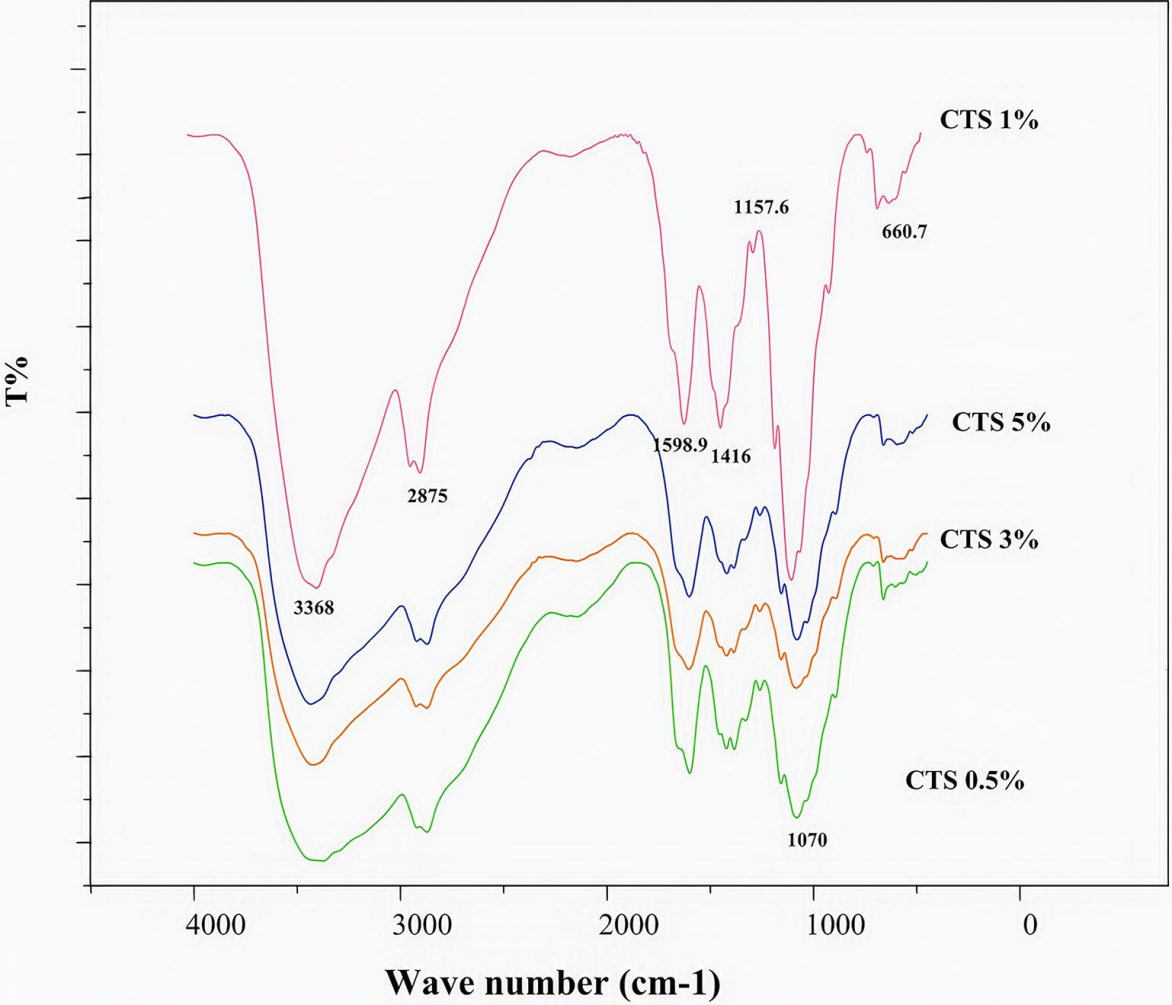
FTIR spectrum of the CH-ZnO NPs with different CH percentages

The results from the different percentages of CH in the CH-ZnO nanocomposite are presented in Fig. 4. The CH with 1% w/v concentration created sharper adsorption peaks and fewer spectrum interactions, showing the best CH concentration in this nanocomposite.

**Fig. 5:**
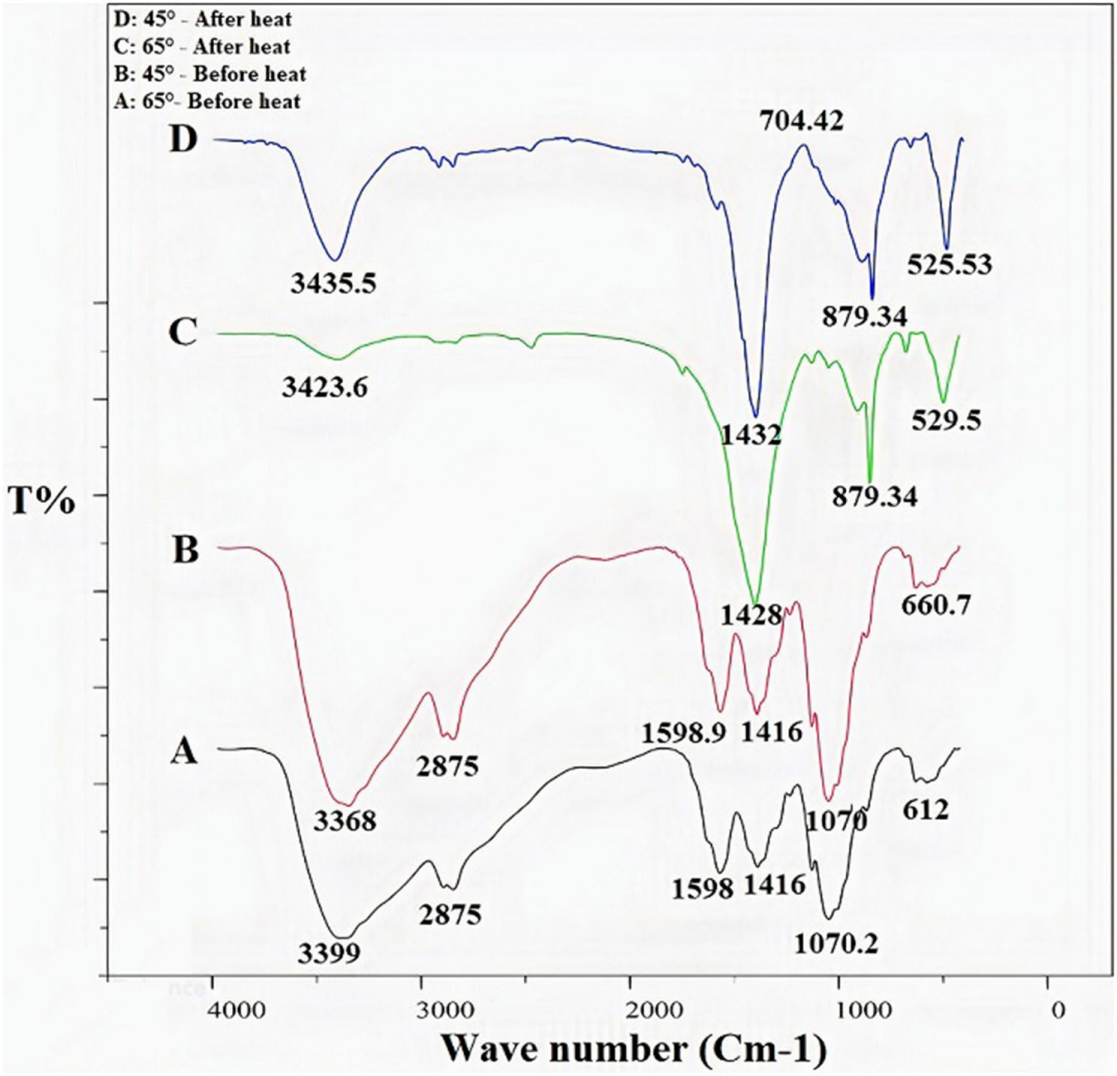
FTIR spectrum of the CH-ZnO NPs at (**A**) 65 ° C before the insertion into the electrical furnace, (**B**) at 45 ° C before the electrical furnace, (**C**) at 65 ° C after the electrical furnace, and (**D**) at 45° C after the electrical furnace

The absorption spectra of the synthesis composite at two temperatures of 45 and 65 ° C before and after insertion into the electrical furnace and 700 ° C temperature are demonstrated in Fig. 5. The absorption spectra of this composite at two temperatures of 45 and 65 ° C did not show a significant difference (A and B lines). After placing the samples in the furnace at 700 °C, the adsorption peaks were sharpened significantly due to evaporating moisture and impurities, which showed the unwanted chemical reactions were eliminated, and the interactions were reduced. Since the adsorption peak at 65 °C was slightly higher than 45 °C, the percent of 1% CH in the composition was recorded as the optimal percentage of CH in the CH-ZnO nanocomposite.

#### XRD pattern

**Fig. 6:**
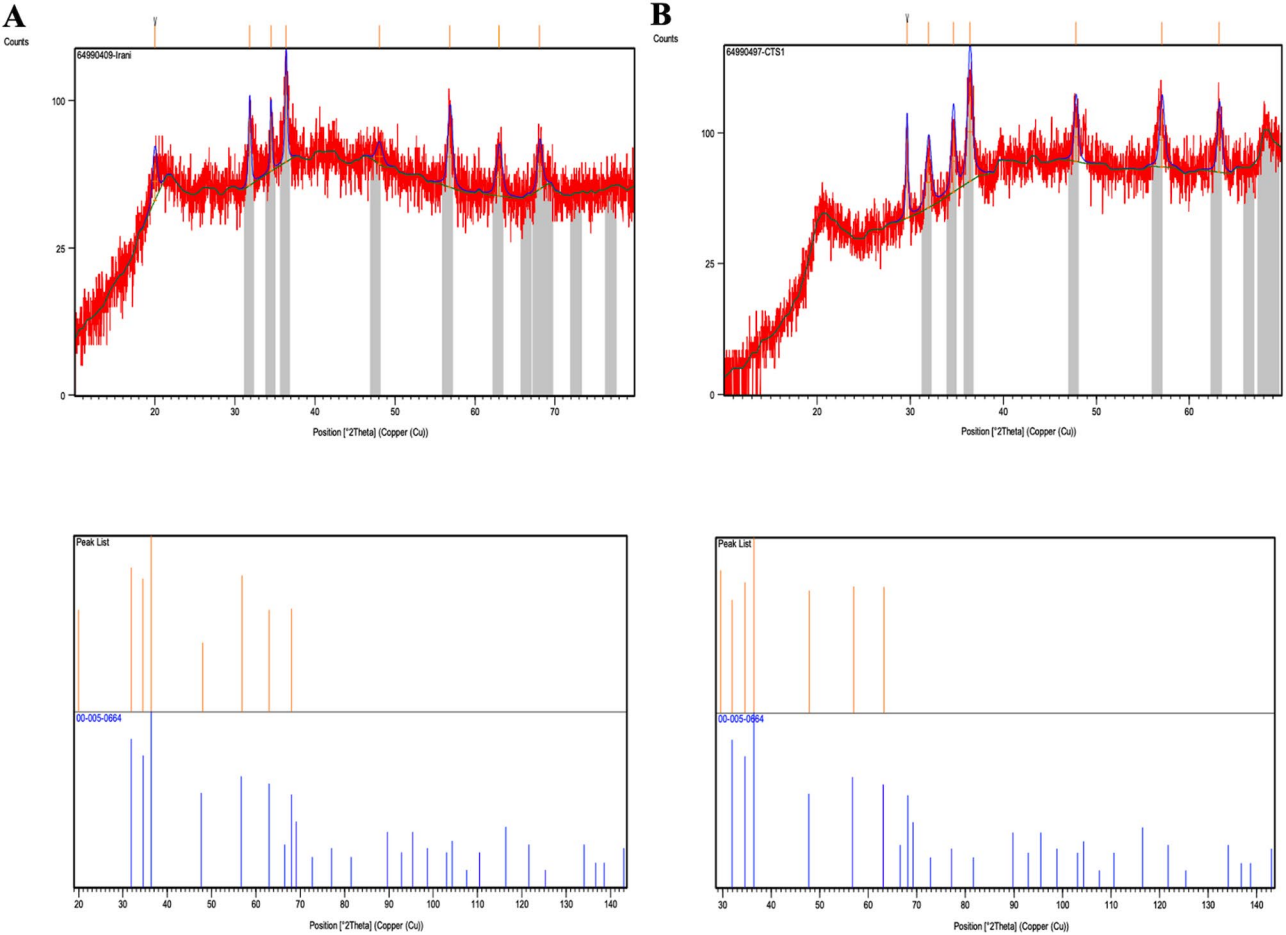
Comparing the diffraction spectrum of (**A**) the CH NPs, (**B**) diffraction spectrum of CH-ZnO NPs

The crystal structure of CH (A) and CH-ZnO (B) in the range of 10 to 80 degrees is depicted in Fig. 6. The normal CH peaks appeared from 10 °C onwards, and a stronger peak appeared at 20.55 °C. The peaks were weakened in the CH-ZnO XRD pattern.

At 32.5, 36.55, 47.69, 56.93, 63.13, and 68.03 °C in the CH-ZnO nanocomposite, XRD peaks were clearer and stronger, indicating (100), (002), (101), (110), (103) and (112) which was related to the hexagonal structure of ZnO plates. The whole XRD peaks confirmed the hexagonal structure of ZnO and the successful formation of the CH-ZnO nanocomposite.

**Fig. 7:**
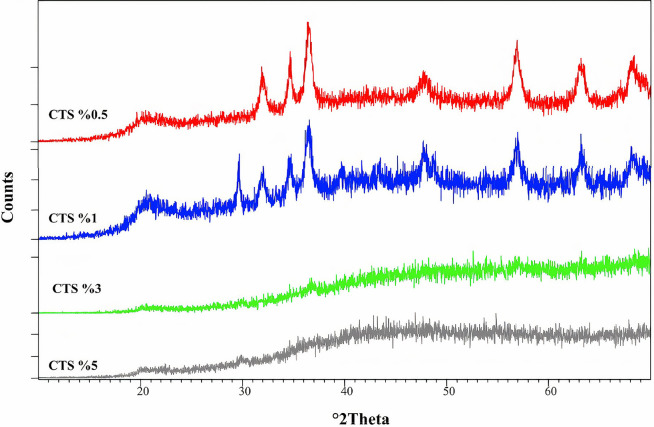
XDR spectrum of the CH-ZnO NPs with different CH percentages

The result of CH diffraction spectra in different percentages is shown in Fig. 7. Two experimental samples containing 3 and 5% CH showed a particular pattern of the amorphous phase, while in 0.5 and 1% CH, the diffraction spectra’ peak angles became larger. Since slightly fewer interactions were seen in the diffraction spectrum of 1% of CH, it was selected as the optimal percentage of CH in the CH-ZnO composite. This result, for the second time, confirmed 1% CH as the optimal percentage.

#### TEM analysis

**Fig. 8:**
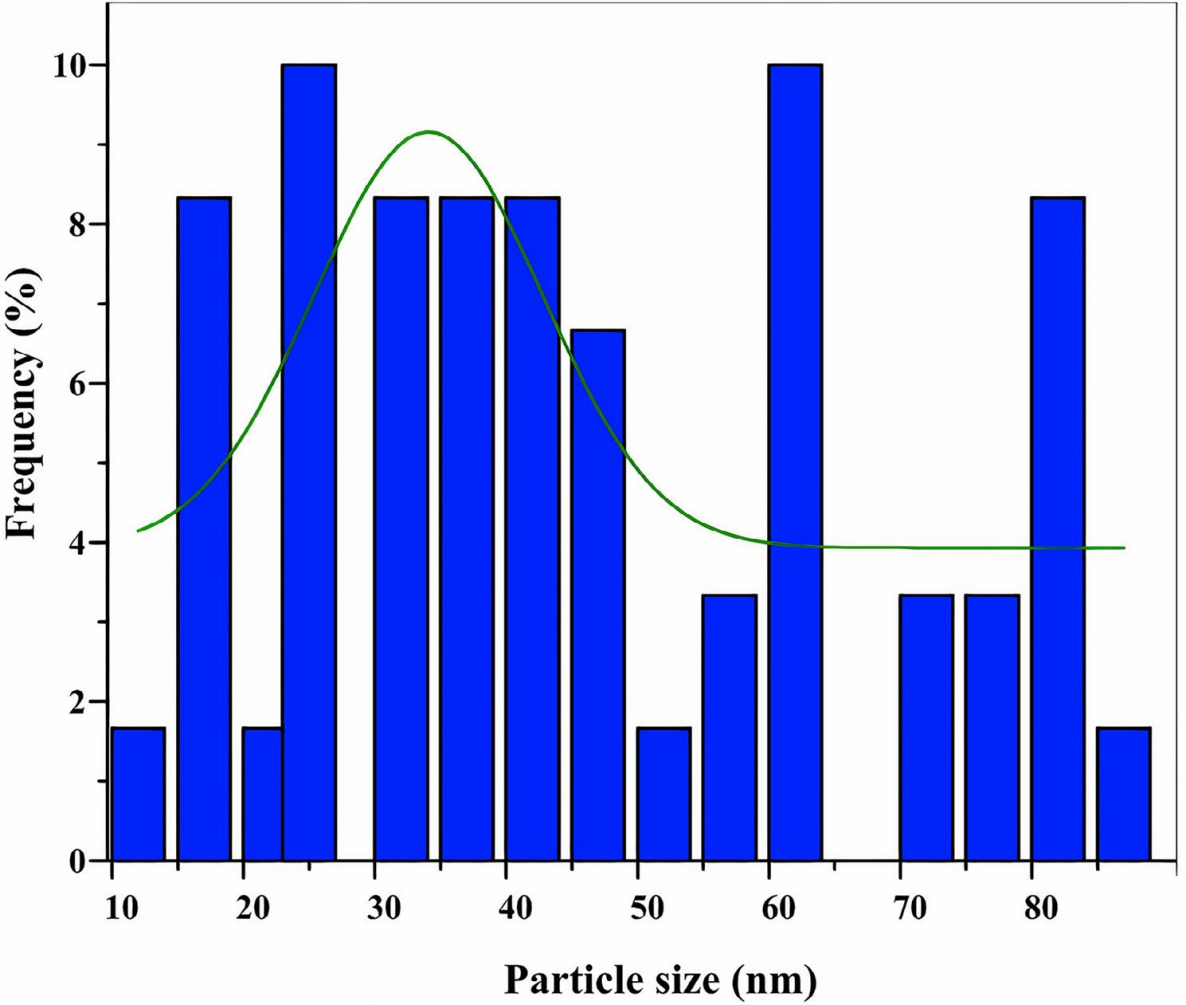
TEM analysis of the CH-ZnO NPs (scale bar 200 and 400 nm)

According to the result of TEM photographs of CH-ZnO nanocomposite, which presented the size and morphology of nanoparticles, nanoparticles within the size range of 35–40 nanometers in spherical shapes emerged as the most prevalent and frequently observed entities (Fig. 8).

### Antibacterial characteristics

#### Planktonic phase (comparison of Coated and CH-ZnO NPs incorporated lollipops)

The effectiveness of three study groups, including two groups of lollipops coated with 2 (group 1) and 4 ml (group 2) of the NPs solution and a group of the lollipops incorporated with 0.7 ml (group 3) CH-ZnO nanocomposite, in planktonic phase against *S. mutans* and *L. acidophilus* was analyzed. CHX was used as the positive control group, and the lollipops with no NPs were used as the negative control group.

#### Comparison of bacterial count between experimental groups

The results of comparing the colony count of study groups are demonstrated in Table 3. The Kolmogorov–Smirnov test revealed the normal distribution of data (P > 0.05). The mean colony counts of the experimental groups 1, 2, and 3 for *S. mutans* bacteria were 1.11 × 107 (5.43 × 106), 4.82 × 106 (5.20 × 106), and 1.24 × 108 (5.48 × 106) CFUs, respectively. For *L. acidophilus*, the mean colony counts of the experimental groups 1, 2, and 3 were 3.56 × 105 (5.28 × 105), 2.26 × 103 (4.34 × 103), 1.40 × 108 (0.00 × 100), respectively. Based on One-way ANOVA, a significant difference was reported between the study groups in both bacteria (P < 0.001). The result of the posthoc test showed that the colony counts of group 1 (lollipops coated with 2 ml of CH-ZnO NPs) were higher than group 2 (lollipops coated with 4 ml of CH-ZnO NPs) for both evaluated bacteria with no significant. However, the antibacterial effect of group 3 (lollipops incorporated with 0.7 ml of CH-ZnO nanocomposite) in the planktonic phase, was significantly lower than two other study groups for both mentioned bacteria (p < 0.001).

**Table 3:** The comparison of colony counts between the experimental lollipops and control groups

Bacteria	Experimental groups	N	Mean (standard deviation)	Comparisons		
				With negative control group	With Chlorhexidine	Between experimental groups
*Streptococcus mutans*	1	5	1.11 × 10^7^ (5.43 × 10^6^)	P < 0.001 ^†^ T = 57.2	P = 0.010 ^†^ T = 4.56	P < 0.001^*^ F = 919.6
	2	5	4.82 × 10^6^ (5.20 × 10^6^)	P < 0.001 ^†^ T = 62.5	P = 0.107 T = 2.07	
	3	5	1.24 × 10^8^ (5.48 × 10^6^)	P = 0.003 ^†^ T = 6.53	P < 0.001 ^†^ T = 54.70	
*Lactobacillus acidophilus*	1	5	3.56 × 10^5^ (5.28 × 10^5^)	P < 0.001 ^†^ T = 634.1	P = 0.206 T = 1.51	P < 0.001^*^ F = 3509
	2	5	2.26 × 10^3^ (4.34 × 10^3^)	P < 0.001 ^†^ T = 77205.7	P = 0.309 T = 1.16	
	3	5	1.40 × 10^8^ (0.00 × 10^0^)			

^*^Statistical significance difference using one-way ANOVA

^†^Statistical significance difference using one- sample *t*-test

#### Comparison of bacterial count between experimental groups and CHX (positive control group)

The results of comparing the bacterial count of study groups with CHX were presented in Table 3. Against *S. mutans* bacteria, the bacterial count of groups 1 and 3 were significantly higher than CHX (P = 0.010 and P < 0.001, respectively). Group 2 showed no significant difference with the positive control group of CHX (P = 0.107).

Against *L. acidophilus*, both of the coated lollipops (groups 1 and 2) had no significant differences with the positive control group (p = 0.206 and p = 0.309).

#### Comparison of bacterial count between experimental groups and lollipops with no NPs (negative control group)

In comparison to the negative control group with 1.5 × 10^8^ bacteria, groups 1 and 2 had a significantly lower *S. mutans* and *L. acidophilus* count (P < 0.001). Group 3 had a significantly lower *S. mutans* count than the lollipop with no NPs, but since the bacteria count for *L. acidophilus* was 1.4 × 10^8^, no statistical test was performed (Table 3).

#### Biofilm phase (comparison of coated and CH-ZnO NPs incorporated lollipops lollipops)

Since none of the study groups could reduce the bacterial colony in the biofilm phase, the antibacterial effects of lollipops with and without CH-ZnO nanocomposite were comparable. The CHX mouthwash, as the positive control group, reduced both bacteria’s biofilms by 80%.

## Discussion

Coated lollipops reduced *S. mutans*, and *L. acidophilus* colonies more efficiently in the planktonic phase. Compared to CHX mouthwash, the coated lollipops with 4 ml NPs against *S. mutans* and both coated ones in contact with *L. acidophilus*, showed comparable cell count. The lower bacterial count of both evaluated bacteria in contact with coated lollipops compared with incorporated ones and the lack of significant difference between the two coated lollipops represented the potential advantages of coated lollipops with CH-ZnO NPs against two evaluated cariogenic bacteria.

In the biofilm phase, none of the experimental lollipops could reduce *S. mutans* and *L. acidophilus*. It may be related to the low solubility of the lollipops in solid form. The dissolution of lollipops in saliva in the oral environment may affect the biofilm formation, which needs clinical studies to confirm.

One of the main problems with antimicrobial materials used in the oral cavity is that they are easily washed out by saliva. Polysaccharides such as CH with muco-adhesive properties remain in the oral environment for an extended time and release their therapeutic agents. The nano-sized CH provides better muco-adhesive and absorption properties than conventional CH [6]. Therefore, we used the nano form of CH-ZnO nanocomposite particles.

Adding 1% of CH to ZnO in CH-ZnO nanocomposite showed favorable outcomes in the aspect of lower interactions in FTIR spectroscopy and XRD pattern evaluation. The were synthesized through the co-precipitation/simultaneous precipitation method, one of the easiest and oldest methods for synthesizing NPs [35].

Dental caries remains one of the most common infectious diseases and a major burden on healthcare systems [1, 36–38]. It is a multifactorial, sugar-driven disease, with *S. mutans* as the main colonizer and an etiological role in caries development [36, 39]. Antibacterial advantages of CH against cariogenic bacteria have been shown in several studies [20, 40–42]. Antimicrobial photodynamic therapy using Emodin-CH NPs has been reported to inhibit significantly biofilm formation and reduce the *S. mutans* virulence potency [43]. Incorporating antimicrobial agents in the form of edibles such as chewing gums and lollipops may be considered an attractive method for delivering anticaries effects, especially for children at higher risk of dental caries [32]. Therefore, we formulated the experimental lollipops containing CH-ZnO NPs.

It should be considered that no flavor and artificial colors were used in the formulation of lollipops to evaluate the only antibacterial effects of the main ingredients of CH-ZnO nanocomposite. At the same time, some authors, such as Hu et al. [14], incorporated them into the lollipops before evaluating their antibacterial properties. These ingredients might be added to the lollipop’s formulation in future evaluation phases of our research.

There are limited data on literature about the addition of antimicrobial agents into the lollipops, in which licorice root extract was used to make sugar-free herbal lollipops. This herb provides antibacterial properties, and the lollipops containing this agent significantly decreased oral cariogenic bacteria in previous studies [11, 13, 14]. Almaz et al. [11] demonstrated that an herbal lollipop containing licorice root extract could decrease salivary S. mutans count in 5–11 years-old children. In Hu et al. [14] study, MIC (minimum inhibitory concentration) of lollipops containing 7 to 15 mg of the licorice root extract in the oral cavity was around 25 times higher than the MIC against the major cariogenic bacteria ranging from 13 to 40 mg/ml. In addition to lollipops, chitosan has been added to chewing gums, reducing oral bacteria significantly [20, 21, 44]. However, the participants in those studies were all adults. Since children are more likely to swallow chewing gum, lollipops may be a more suitable method of administering chitosan.

In one of the experimental groups (group 3) of this study, 0.7 ml of CH-ZnO nanocomposite was mixed with the base of the lollipop (liquid isomalt) at a high temperature (more than 120 degrees). Since Kumer et al. [45] confirmed the thermal resistance of CH-ZnO nanocomposite and no destruction of its matrix up to 460 degrees, there was no concern that high temperature may negatively affect the CH-ZnO nanocomposite. However, due to the lack of authentic evidence about the negative effects of high temperature on the structure and antibacterial properties of CH and its composites, coated lollipops with 2 and 4 ml of NPs were also made. In these groups, the cooled lollipops were coated with CH-ZnO NPs containing solution to compare with incorporated ones to evaluate the potential effects of heat on antibacterial properties.

According to the results of this study in the planktonic phase, both coated lollipops reduced colonies of *S. mutans* and *L. acidophilus* bacteria more than mixed ones. It may be related to several factors. For instance, the heat in the CH-ZnO NPs incorporated lollipops may have a detrimental effect on CH-ZnO NPs and reduced antibacterial effects. Also, the amount of CH-ZnO NPs on the surface of lollipops and their release from the surface may be more than inside the lollipops structure. In addition, the concentration of CH-ZnO NPs in the incorporated lollipops was 0.7 ml which was lower than 2 and 4 ml used in the coated ones.

Xanthan was used to prepare the coating solution to increase the viscosity and improve the lollipops’ adhesion to the isomalt base. It has also been used in the drug delivery system to increase the duration of release from the drug system [46]. Huang et al. [47] used this material combined with CH, and carboxymethyl-modified chitosan (NOCC) to produce an injectable hydrogel with various properties, such as accelerating wound healing. Adding this material to the lollipops could be helpful in gradually releasing NPs in the oral environment and increasing the antibacterial agent contact with dental surfaces. However, further studies are needed to confirm this hypothesis.

Since demineralization in enamel and dentin begins at pH values of 5.5 and 6.4, respectively, anti-caries agents should be adjusted to neutral or alkaline pH levels [48, 49]. The coating solution of lollipops in the present research was set to a pH of 10.0 and prepared in alkaline form. No acidic ingredients were used to reduce the possibility of creating an acidic environment after the lollipop consumption, which is favorable for acidogenic and caries-induced bacteria. Based on this study’s results, the coated lollipops with CH-ZnO NPs reduced two evaluated caries-induced colonies in the planktonic phase. The lack of significant difference between CHX and lollipops coated with 4 mL NPs in contact with *S. mutans* and comparable results among CHX and both coated lollipops against *L. acidophilus* confirmed the great antibacterial effects of CH-ZnO nanocomposite containing lollipops. The reduction in both bacterial colonies in coated lollipops compared with the negative control group (lollipops with no NPs) determined the antibacterial effects were related to the only NPs and other ingredients of lollipops did not produce antibacterial effects. By the present study results, Hayashi et al. [20] showed the antibacterial properties of CH xylitol-based gums in 50 patients by significantly reducing *S. mutans*, total *streptococci*, and total bacteria counts. An 80% drop in salivary S. mutans levels was seen in their study, even up to one hour after consuming chitosan-containing chewing gum. In our in vitro study, however, the *L. acidophilus* population was also evaluated, and their decline in the planktonic phase was verified.

The antibacterial effects of CH and ZnO are related to interfering with the bacterial cell wall and causing cell disruption [40], creating reactive oxygen species and damaging cell membranes [50, 51]. Since combining CH with inorganic metal oxide has been shown to improve antibacterial properties, in this study, the combination of CH and ZnO NPs has been used. This composite presented favorable outcomes in contact with bacteria, including *Escherichia coli*, *Pseudomonas aeruginosa*, *Staphylococcus aureus*, *Bacillus subtilis* and fungi such as *Candida Albicans* [27]. Moreover, Shim and Haldorai [52] showed that 92/99% of *E. coli* cells were deactivated after 24 incubations with CH-ZnO NPs.

Despite the lack of studies evaluating the effect of this nanocomposite in food products, there is limited studies that have investigated the antibacterial effects of CH and ZnO alone or in the composite form in dental materials. For instance, Sodagar et al. [41] added CH NPs of three different concentrations, 1%, 5%, and 10%, to orthodontic composite and found that these particles induced an antibacterial activity in the resin composite. In another study, Rajabnia et al. [42] found that sealants containing 2–5% CH show an antimicrobial property that is intensified by increasing the CH concentration. In this study’s agreement, by increasing the concentration of CH-ZnO NPs from 0.7 (in CH-ZnO NPs incorporated lollipops) to 4 ml (in group 2 of coated lollipops), the antibacterial effect was improved. The same results were reported by Aliasghari et al. [24] and Sodagar et al. [41]. However, confirming the relation of concentration and antibacterial effects is not the aim of this study and this statement needs more research in future investigations using different concentrations of CH-ZnO NPs.

CH was also used in kinds of toothpaste formulations to protect mineralized surfaces [53–55]. Afrasiabi et al. found that a CH hydrogel-based ZnO nanocomposite decreased *S. mutans* virulence by lowering metabolic activity and biofilm formation [56].

This was a preliminary study evaluating the potential antibacterial effects of CH-ZnO nanocomposite in lollipops; therefore, some limitations should be considered. This study assessed the antibacterial effects through bacterial counts and biofilm formations. More microbial tests and further clinical studies are needed to confirm the antibacterial properties of edible materials. Since no study has been reported on the toxicity of CH and its composites yet, and it has also been used vastly in reducing cholesterol and weight loss [57–60], there is no concern about its application in food products. However, their biocompatibility should be confirmed in contact with oral mucosa and gastrointestinal cells.

Finally, it must be noted that using lollipops or gums containing antibacterial agents should be prescribed by clinicians, especially for those at high risk of caries and children, as a supplement and efficient method of preventing dental caries [14]. In this way, the candies and lollipops can stimulate saliva, and the advantages of clearance of acidogenic bacteria and their products were added to the antibacterial agents’ positive effects.

In conclusion, the lollipops coated with 2 and 4 ml of CH-ZnO NPs showed good antibacterial efficacy in the planktonic phase against two cariogenic microorganisms, *S. mutans*, and *L. acidophilus*, whereas those incorporated with NPs were not effective as well as the coated ones. As a result, this experimental lollipop may be a valuable supplement for lowering cariogenic bacterial counts, particularly in people at high risk of dental caries. However, further studies are needed to demonstrate their efficacy against biofilm formation in clinical settings.

## Data Availability

The datasets used and/or analysed during the current study available from the corresponding author on reasonable request.
